# Development of a short form and scoring algorithm from the validated actionable bladder symptom screening tool

**DOI:** 10.1186/1471-2377-13-78

**Published:** 2013-07-09

**Authors:** David Bates, Jack Burks, Denise Globe, Manuel Signori, Stacie Hudgens, Pierre Denys, Scott MacDiarmid, Victor Nitti, Ib Odderson, Amy Perrin Ross, Michael Chancellor

**Affiliations:** 1 Department Neurology, RVI, University of Newcastle, Newcastle on Tyne NE1 4LP, England; 2 Burks and Associates, 650 John Fremont Dr., Reno, NV 89509, USA; 3 Allergan, Inc., 2525 Dupont Drive, Irvine, CA 92612, USA; 4 Adelphi Values, 133 Portland Street, Boston MA 02114, USA; 5 Raymond Poincaré Hospital, Université de Versailles, Saint Quentin UVSQ, 92380, Garches, France; 6 Alliance Urology Specialists, 509 N Elam Avenue, Greensboro, NC 27403, USA; 7 NYU Langone Medical Center, 150 East 32nd Street, New York, NY 10016, USA; 8 Department of Rehabilitation Medicine, University of Washington, Box 356490, 1959 NE Pacific Street, Seattle, WA 98195, USA; 9 Loyola University, 918 Red Fox Lane, Oak Brook, IL 60523, USA; 10 Oakland University William Beaumont School of Medicine, 438 Medical Office Building 3535 W. 13 Mile Road, Royal Oak, MI 48073, USA

**Keywords:** Overactive bladder, Multiple sclerosis, Patient reported outcome, Quality of life, Validation

## Abstract

**Background:**

The majority of multiple sclerosis (MS) patients develop some form of lower urinary tract dysfunction, usually as a result of neurogenic detrusor overactivity (NDO). Patients identify urinary incontinence as one of the worst aspects of this disease. Despite the high prevalence of NDO, urological evaluation and treatment are significantly under-accessed in this population. The objectives of this study were: 1) to adapt the previously validated Actionable Bladder Symptom Screening Tool (ABSST) to a short form for ease and brevity of application in a clinical setting that is clinically meaningful; and 2) to develop a scoring algorithm that would be interpretable in terms of referring/considering precise diagnosis and treatment.

**Methods:**

A US-based, non-randomized, multi-center, stand-alone observational study was conducted to assess the psychometric properties of the ABSST among patients who have MS with and without NDO. Mixed psychometric methods (e.g., classical statistics (*Psychometric theory (3rd ed.).* New York: McGraw-Hill; 1994) and item response methods (*Applying the Rasch Model: Fundamental Measurement in the Human Sciences.* New Jersey: Lawrence Earlbaum Associates; 2001)) were used to evaluate the predictive and clinical validity of the shortened form. The latter included clinicians flagging clinically meaningful items and associated response options which would indicate the need for further evaluation or treatment.

**Results:**

A total of 151 patients, all with MS and with and without NDO, were recruited by 28 clinicians in various US geographical locations. Approximately 41% of patients reported a history of or currently having urinary incontinence and/or urinary urgency. The prediction model across the entire range of classification thresholds was evaluated, plotting the true positive identification rate against the false positive rate (1-Specificity) for various cut scores. In this study, the cut-point or total score of greater than or equal to 6 had a sensitivity of approximately 85%, and specificity of approximately 93% (i.e., 85% patients would warrant being referred to a urologist and 93% of the patients whose symptoms would not warrant urologist referral).

**Conclusions:**

Overall the short form ABSST demonstrated sensitivity and specificity as it maintained the integrity of the longer form tool. Concurrent validity for each subscale as well as predictive and concurrent validity of the total shortened instrument was demonstrated. This instrument provides a new method for assessing bladder problems among MS patients, and may facilitate earlier and more precise diagnosis, treatment, and/or referral to a specialist.

## Background

Multiple sclerosis (MS) is a progressive neurologic disorder that causes demyelination of affected nerves in the central nervous system [1-3]. MS can cause almost any neurological symptom, and it often progresses to cognitive and neurological disabilities in the patient, including a decline in mobility [1]. MS affects women three times as often as it affects men. It affects approximately 1 in 1000 people in the US and is the most common cause of neurological disability in individuals 20–45 years old [4,5]. A majority of MS patients develop some form of lower urinary tract dysfunction due to disconnection between the brainstem and the lower spinal cord [6-8]. In particular, up to 75% of MS patients have neurogenic detrusor overactivity (NDO), a bladder disorder characterized by spontaneous overactivity of the detrusor muscle [9]. Symptoms of this disorder include urinary urgency, urinary frequency, and/or urinary incontinence [2,10]. Incontinence has been identified as one of the worst aspects of the disease from the patient’s perspective [8]. High intravesical pressures may also lead to serious complications, including kidney infection, and reflux, ultimately causing irreversible damage [7]. Additionally, given all of the issues with NDO in MS it is noted that research groups are establishing guidelines for the evaluation of urinary disorders in MS [7,8,11]. Therefore, despite the high prevalence of urinary incontinence, urological evaluation and treatment are significantly under-accessed in this population [10]. This study outlines the development and validation of a shortened form of a new screening tool, the Actionable Bladder Symptom Screening Tool (ABSST), that healthcare providers can use to identify MS patients with urinary incontinence who may be in need of a more precise diagnosis and/or treatment for their urologic symptoms, including referral to a urologist. The objectives of this study were twofold: 1) to adapt the previously validated 17-item ABSST to a short form for ease and brevity of application in a medical setting that is clinically meaningful; and 2) to develop a scoring algorithm that would be interpretable in terms of referring/considering diagnosis and treatment. Novel item response and classical test methods were used to identify optimally performing items for inclusion in the short form assessment.

In view of recent research suggesting that patients and clinicians are more likely to use shorter instruments [12-15], it was decided to develop a short form version of the ABSST that is clinically meaningful from the clinician’s perspective. The development of this short form version of the instrument is described here. In addition, a scoring algorithm was developed for the ABSST, with a identification of a score that would recommend further diagnosis or referral to a urologist. The novel approach of developing the scoring algorithm presented here is based on pivot anchoring as a means of demonstrating clinical meaningfulness.

## Methods

### Multi-site observational study

Data to support the short form adaptation was collected in a US-based, non-randomized, multi-center, stand-alone observational study in male and female patients who have MS with and without NDO. MS patients were recruited through neurology practices to complete patient reported outcome assessments (PRO) (the ABSST and the Overactive Bladder Questionnaire – Short Form (OAB-q-SF)) as well as a demographic and health information form. Written informed consent was needed before the subject could participate in any part of the study. All research involving patient interviews and patient completion of questions were in compliance of the Helsinki Declaration and had full Institutional Review Board (IRB) approval from Copernicus Group (an independent institutional review board organized and operating in compliance with regulations governing institutional review boards set forth in 21 CFR and ICH guidelines, as well as 45 CFR when applicable.) (Protocol # MAP1-11-049). Completed ABSST questionnaires (with patient specific identifying information removed) were shared with the referring clinician and the clinician denoted whether or not referral to a urologist was recommended based on the specific patient’s responses. The presence of NDO was not a requirement for inclusion into the observational study. Details of the design of the multi-site observational study will be reported in a separate publication summarizing the development and validation of the ABSST long form [16].

### Psychometric criteria/methods for inclusion on the ABSST-short form

The psychometric evaluation and subsequent development of the ABSST-Short Form integrated responses on the longer version of the tool collected from both patients and clinicians. A set of predefined statistical criteria was developed and tested to ensure that the resulting shortened tool included clinically meaningful and reliable items. These were as follows:

•Classical statistical methods

Percentage of patient responses at the floor of measurement (lowest response option) less than 50% [17].

Item correlation with the overall scale (e.g., the extent to which the items are related to other items on the scale) greater than 80% [17].

•Item response theory methods

Degree to which patients response as expected (e.g. measurement error) as measured by Rasch Infit Statistics [18] (acceptable criteria between 0.60 and 1.40).

•Clinician perspective

Greater than 50% of clinicians indicated that the item was clinically relevant.

For clinician response, a pivot anchoring approach was used. In this analysis, the 23 recruiting clinicians were asked to review the ABSST, circle the items that were important to them, and indicate the threshold at which they thought the items would indicate a potential bladder problem or be a cause for concern [18,19].

### Classical statistical methods

Individual items were evaluated using two key classical statistical methods: percentage of items at the floor (lowest response option) of measurement and degree to which individual items correlated with the overall scale score. Floor effect refers to a high percentage of patients scoring the lowest score on an individual item. If floor effects are too pronounced, it could interfere with the ability of the instrument to screen patients as experiencing problems. An item was considered to have floor effects if greater than 50% of patients endorsed the lowest category. The degree to which individual items correlated highly with the remaining items on the scale was measured as the magnitude of the correlations ≥ 0.80 [17].

### Item response theory methods - rasch analysis

Individual items of the ABSST were evaluated using the Partial Credit model, an extension of the 1-parameter Rasch model for use with items using multiple response formats (dichotomous, polytomous). A Rasch analysis allows for the accumulation of evidence associated with each response to an item by a group of respondents rather than relying on group level statistics (i.e., classical test theory) [20]. More specifically, this particular model allows for the joint estimation of symptom severity (item difficulty) and person’s level of severity (person ability). The underlying assumptions for this item analysis are: 1) local independence and 2) unidimensionality. Local independence is evidence that items are conditionally independent of each other (i.e., each item measures a unique symptom). Unidimensionality is evidence that items on a scale measure one underlying trait (e.g., overactive bladder (OAB) symptoms). In the case of symptom measures, we assume the underlying trait as a hierarchical symptom structure which may, in fact, be considered multidimensional. The term unidimensionality, therefore, indicates the underlying symptomology of the condition.

The ability of the ABSST items to reflect an underlying latent construct was assessed by performing item fit analysis. Infit and outfit statistics compare the actual responses on the survey with responses predicted along the range of OAB severity. Acceptable values range from 0.60 – 1.40 for questions with rating scale response options [18]. Items that do not fit the Rasch model may be measuring domains other than the domain of interest or may elicit atypical responses (e.g., endorsement of a high severity symptom by persons with few symptoms). Two item fit statistics were calculated: mean-square infit and mean-square outfit. High infit reflects the tendency of the item to elicit unexpected responses among respondents whose level on the measure approximates the difficulty of the item. High outfit reflects the tendency of the item to elicit unexpected responses among respondents whose level on the measure is above or below the difficulty of the item. Mean-square infit and outfit statistics ≥ 1.40 indicate significant item misfit.

As a further test of local independence of the ABSST domains, individual items were evaluated under the Rasch model using the person separation and reliability, analogous to the Kuder-Richardson Formula-20 (KR-20) [20-23]. The KR-20 is defined as a measure of reliability for dichotomous response choices. Values above 0.90 indicate homogeneity of responses. Under the Rasch model, KR-20 is used as each response category has a hypothesized probability of 0.50 of endorsement to the adjacent category. The item and person separation indices estimate the separation of persons and items on the underlying latent variable. Rasch measurement allows for the additional analysis of both items and persons distributed along the same linear continuum (trait). In order to evaluate whether each of the domain items covers the continuum, items must be sufficiently separated in terms of their item difficulty (which can represent the severity of the underlying trait). The threshold for separation is an index of 2.0 and an associated separation reliability of 0.80 [22]. Rasch person reliability estimates allow for evaluation of whether the items appropriately estimate a person’s symptom severity on the underlying trait. When reliability falls below the customarily accepted threshold of 0.70, it indicates that patients may be experiencing symptoms that the measure does not cover (i.e., construct deficiency) [22]. Rasch item reliability estimates the item severity range (i.e., Does the severity scale associated with the items cover the distribution of severity?); which is considered acceptable if ≥ 0.70 as well [22].

#### Pivot anchoring

In order to create a clinically useful ABSST, a pivot anchoring analysis was conducted utilizing both novel and classical test theories to identify optimal items. All 28 recruiting clinicians were asked to review the ABSST and circle which items were important to them in their clinical decision making, and at what threshold they thought the items would indicate a potential bladder problem or would be a cause for concern. Twenty-three responses were received and used in the pivot anchoring analysis.

A scoring method was developed which included both the clinician and patient responses. First, the clinicians referring patients to the quantitative study were surveyed on attributes of the patient population relative to the instrument. Each clinician was then asked to specify the lowest rating on each of the 16 items which would indicate a clinically meaningful potential bladder problem. Based on the clinicians’ ratings of the items, each patient’s response was then denoted by either a 1 (indicating a potential bladder problem) or a 0 (indicating no potential bladder problem). This scoring algorithm was then tested using classical test theory and item response theory methods (described in Pivot Anchoring and Predictive Validity sections).

#### Predictive validity

Logistic regression was used to determine the predictive validity of the ABSST total score to identify patients that would receive a recommendation to see a urologist. The predictive value was based upon a clinician rating of ‘Yes’ or ‘No’ on whether they would recommend a patient to see a urologist based upon the patient’s responses to the ABSST. Results from logistic regression models testing different cut-points predicting the recommendation were summarized with odds ratio, sensitivity and specificity, Positive predictive value (PPV), Negative predictive value (NPV), percent who warranted referral, and area under the receiver operating curve (ROC). The odds ratio was defined as those MS patients more likely to be referred to a urologist than not. The sensitivity refers to those results that are true results (e.g. would refer to a urologist) while specificity refers to those results that are truly negative results (e.g. would NOT refer to a urologist). PPV refers to proportion of positive test results that are true positives (e.g. proportion of patients who would be referred to a urologist are warranted to be referred) while the NPV refers to the proportion of negative results that are true negatives (e.g. the proportion of patients who would NOT be referred to a urologist are NOT warranted to be referred). The percent of those patients who warrant referral is the percentage of patients classified as either a referral being warranted to a urologist or not. The area under the ROC refers to the ability to classify those who would warrant or would not warrant being referred to a urologist [24].

## Results

### Patient population

A total of 151 patients, all with MS and with and without NDO, were recruited by 28 clinicians in various US geographical locations to participate in the study. Patients had a mean age of 48.2 (SD 12.11) years, with age ranging from 22 to 80 years of age. Patients had been diagnosed with MS an average of 9.1 (SD 7.24) years. Approximately 70% of patients described the severity of their MS symptoms over the past 6 months as mild, 23.8% described their symptoms as moderate, 1.3% described their symptoms as severe , and 4.6% described them as none/not applicable (4.6%). Severities were captured using patient self-report in a demographic form. Patients were asked how severe their MS symptoms were in the past 6 months on a 0–3 rating scale (0=Not applicable / None, 1 = Mild, 2 =Moderate (uses aides to walk), 3 = Severe (uses wheel chair sometimes)). Approximately 41% of patients reported having a history of or currently having urinary incontinence and/or urinary urgency.

### Short form development

Table 1 outlines the results of the predefined statistical criteria that were developed to ensure that clinically meaningful and reliable items were included (please refer to Section 2.2). Eight items (plus the final “Yes/No” item) were identified from the original 17-item ABSST. Boxes marked with an (X) represent those items that met the patient level criteria. The black boxes indicate those items that met the clinician level analysis or statistical criteria. The final row in the table indicates important items from the pivot anchoring analysis. For example, on Item 1 (Urinate right away), if a clinician saw that a patient responded with a “4” (All of the time) then it would be a cause for concern and possibly referral to a urologist. Item 17 is the “Yes/No” item asking patients if they would seek help for their bladder issues. Please see Additional file 1: Table S1 for the ABSST short form.

**Table 1:** Clinician and patient short form results

**Criteria**	**Item number**								
	**Item 1**	**Item 3**	**Item 7**	**Item 8**	**Item 9**	**Item 10**	**Item 11**	**Item 13**	**Item 17**
	**(Urinate**	**(Urinary**	**(Need to**	**(Wake to**	**(Number of**	**(Activities with friends**	**(Ability to**	**(Embarrassed)**	**(Receive**
	**right away)**	**accidents/**	**urinate right**	**urinate)**	**times urinated)**	**and family affected)**	**work**		**help)**
		**leakage)**	**away)**				**affected)**		
Floor effect less than 50%	X		X	X	X				N/A
Item Correlation with Scale greater than 80%	X		X			X		X	N/A
Infit statistics between 0.80 and 1.20	X	X	X	X		X	X		N/A
Met all statistical inclusion criteria	++		++						++
Greater than 50% of clinicians considered clinically meaningful		++		++		++	++		
**Rating Clinician reported as critical for screening (General MS Patient)**	4	3 or 4	4	4	3 or 4	3 or 4	3 or 4	3 or 4	1

X= Patient level analysis.

++= Clinician level analysis.

#### Classical statistical results

Evaluation of the classical statistical methods, floor effect and item correlation statistics, indicated that items 1, 7, 8, 9, 10, and 13 met one or both criteria for inclusion into the short form. More specifically, items 1, 7, 8, and 9 met the criteria for inclusion into the short form based on floor effect less than 50%; and items 1, 7, 10 and 13 met the criteria for inclusion into the short form based on item correlation with scale greater than 80%.

#### Item response theory Rasch analysis results

Based on the criteria detailed in the methods section, each of the items on the ABSST demonstrated the evidenced person reliabilities ≥ 0.70. Fit statistics indicated that 9 item responses were within the expected criteria (Table 2). However, items 2, 4–6 and 14–16 scored outside of the acceptable ranges, demonstrating less than desirable infit or outfit statistics. For example, Item 16 was shown to not directly measure Impact of Symptom Intensity as it scored an infit statistic of 2.68 and an outfit of 2.57, both of which are outside of the threshold of 1.6 – 2.4. This result suggests poor fit to the Rasch model. Similarly, Items 2, 4, 5, 6, 14, and 15 fell outside of the acceptable range as previously described.

**Table 2:** Infit and outfit statistics by item

	**Symptom**	**Infit MNSQ**^**1**^	**Outfit MNSQ**
**Bladder Symptoms Intensity** (Separation=9.74; Reliability=0.99)	**ITEM 9 (Number of times urinated)**	**1.23**	**0.79**
	**ITEM 8 (Wake to urinate)**	**0.98**	**0.97**
	**ITEM 7 (Need to urinate right away)**	**0.92**	**0.89**
**Impact of Bladder Symptoms Intensity** (Separation=3.77; Reliability=0.93)	ITEM 16 (Depressed)	2.68	2.57
	**ITEM 10 (Activities with friends and family affected**	**0.43**	**1.13**
	**ITEM 11 (Ability to work affected)**	**0.52**	**1.11**
	**ITEM 13 (Embarrassed)**	**0.64**	**0.75**
	ITEM 15 (Worried)	0.42	0.75
	ITEM 14 (Frustrated)	0.52	0.7
**Bladder Symptoms Frequency** (Separation=4.40; Reliability=0.95)	**ITEM 3(Urinary accidents/leakage)**	**1.14**	**1.01**
	**ITEM 1(Urinate right away)**	**0.94**	**1.01**
**Bladder Coping Strategies Frequency** (Separation=4.85; Reliability=0.96)	ITEM 4 (Use of leakage protection in the day)	1.38	0.96
	ITEM 5 (Use of leakage protection at night)	1.23	1.38
	ITEM 6 (Limit amount of fluid)	0.78	0.82
	ITEM 2 (Make sure know where bathrooms are)	0.67	0.64

^1^Infit and outfit statistics compare the actual responses on the survey with responses predicted along the range of OAB severity. Acceptable values range from 0.60 – 1.40 for questions with rating scale response options [18].

#### Predictive validity

Results from logistic regression models testing different cut-points predicting the recommendation to refer to a urologist are summarized in Table 3. The results of the odds ratio, sensitivity and specificity, PPV, NPV, and percent correctly classified suggest a raw score of 6 or more for further evaluation from a urologist. The overall ABSST Total Score c-statistic was equal to 0.928 and the logistic regression model was robust (Hosmer and Lemesow Goodness-of-Fit Test (p=0.5180)).

**Table 3:** Performance of the revised ABSST total score at various cut-points* predicting Clinician’s Urologist Referral

**Cut-Point±**	**Odds**	**Sensitivity**^**2**^	**Specificity**^**3**^	**Positive**	**Negative**	**% Warranting**	**c-statistic**^**7**^
	**ratio**^**1**^					**referral**^**6**^	
				**Predictive value (%)**^**4**^	**Predictive value (%)**^**5**^		
>= 21	.	2.04	100.00	100.0	68.0	68.2	0.510
>= 9	55.53	69.39	96.08	89.5	86.7	87.4	0.827
>= 6	81.43	85.71	93.14	85.7	93.1	90.7	0.894
>= 4	33.54	93.88	68.63	59.0	95.9	76.8	0.813
>= 1	8.28	97.96	14.71	35.6	93.8	41.7	0.563

* Only quartiles and key cut points are displayed in the table.

The cut point is defined as different total raw scores on the ABSST ranging from 0 to 24.

^1^ The odds ratio was defined as those MS patients more likely to be referred to a urologist than not. Values > 1 indicated that the patient is that many more times (for example 8.28) likely to be referred to a urologist.

^2^ The sensitivity refers to those results that are true results (e.g. would refer to a urologist). Minimum criteria was ≥0.75.

^3^ Specificity refers to those results that are truly negative results (e.g. would NOT refer to a urologist). Minimum criteria was ≥0.80.

^4^ PPV refers to proportion of positive test results that are true positives (e.g. proportion of patients who would warrant a referral to a urologist). Values closer to 100% approximates higher proportion of true positives.

^5^ NPV refers to the proportion of negative results that are true negatives (e.g. the proportion of patients who would NOT warrant a referral to a urologist are not referred). Values closer to 100% approximates higher proportions of true negatives.

^6^ The percent of classified patients is the percentage of patients who are warranted to be referred to a urologist or not.

^7^ The c-statistic is the area under the ROC curve. Values closer to 1 approximate a perfect model.

**Figure 1:**
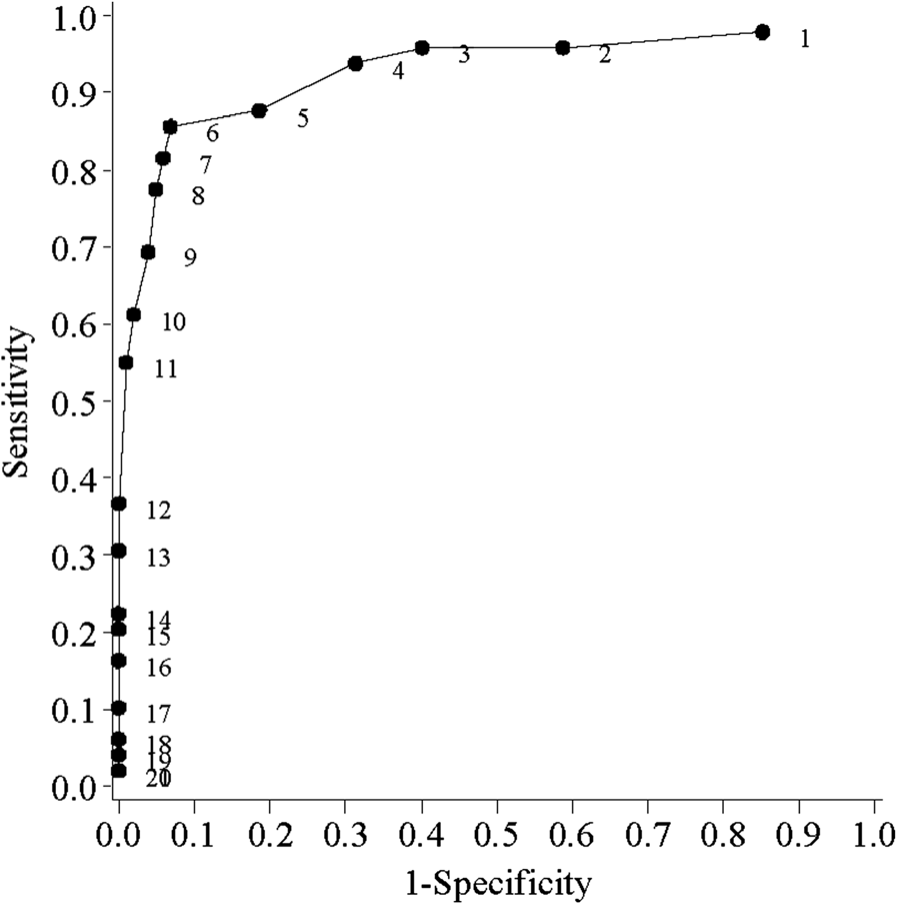
ROC Curve of the ABSST Total Score at Cut-Points Predicting Referral to a Urologist.

The performance of the ABSST total score predicting clinician’s referral to a urologist is presented in Figure 1 via the ROC curve. Figure 1 displays how different cut-points on the ABSST total score affect sensitivity and specificity of the prediction for referral to an urologist. The prediction model across the entire range of classification thresholds was evaluated; plotting the true positive identification rate against the false positive rate (1-Specificity) for various cut scores. In this study, the cut-point of greater than or equal to a raw score of 6 had a sensitivity of 85.7%, and specificity of 93.1% (i.e., 85.7% patients would warrant a referral to an urologist and 93.1% of the patients would not warrant a referral to an urologist).

## Discussion

The goal of this study was to validate the short form Actionable Bladder Symptom Screening Tool (ABSST) as a patient driven, clinically useful measure that is easy to administer and score. The goals were to develop a tool that is sensitive, MS-specific, easy to interpret, easy to use in a clinical setting, and multidimensional, all of which encourage patient-clinician interaction. The unique application of a mixed methods approach was used to select the best items from the long form, using both qualitative and quantitative techniques.

The original, long form ABSST was developed as a de novo measure after it was determined that existing measures did not appropriately assess the symptoms and impacts of NDO on MS patients. The long form of the ABSST is a 17-item instrument (16 items with an additional “Yes/No” item asking patients if they would like to receive help for their bladder problems) that covers three domains: Bladder Symptoms, Coping Strategies, and Impact of Bladder Symptoms. It was developed using established qualitative methods [25,26], including a literature review and clinician input to identify potential symptoms, open-ended concept elicitation interviews with MS patients who have OAB, and face and content validity testing through cognitive interviews (face-to-face debriefing interviews on the ABSST – details to be reported in another publication) with another group of MS patients with OAB. Once the qualitative phase was completed, a US-based, non-randomized, multi-center, stand-alone observational study was carried out to evaluate the measurement properties of the newly developed instrument. Analysis of item completion, item and scale distribution, and predictive validity of the long form ABSST demonstrated strong psychometric properties (details reported in another publication) [17,19,27-30]. The ABSST total score also demonstrated predictive validity, identifying patients who would receive a referral. In order to demonstrate that this screening tool was valid and reliable as a short form, classical test theory and item response theory approaches were taken to ensure that each item included provided the most information, was clinically meaningful, and demonstrated predictive attributes for patient referral to a urologist. Overall, concurrent validity for each subscale as well as predictive and concurrent validity of the total instrument were shown, demonstrating strong psychometric properties.

The simplified scoring method for bladder problem assessment developed here will make it easier for clinicians to identify patients with MS who may have potential problems. The approach used to develop the scoring algorithm adheres to the FDA guidance, is psychometrically valid, and appropriately utilizes pivot anchoring [31], which has been widely used in the interpretation of clinically meaningful points along a categorical continuum. This methodology allowed for synthesis of meaningful cut-points along the continuum where patients are likely to demonstrate urinary problems, as reported by clinicians. This mixed statistical methods approach to item reduction and scoring optimizes selection of items for making sound clinical decisions based on clinicians’ assessment of the need for patient referral. These data, when subjected to predictive validity calculations, provided very strong results indicating that clinicians could use it to refer patients appropriately.

This study has several limitations. First, the ABSST is a screening tool and was not designed for diagnostic purposes. Second, although it was identified as being clinically meaningful, the relevance in overall clinical practice has not yet been tested. Lastly, because of the error range on the tool itself, it is possible that some patients’ symptoms may go untreated (under-sensitive results) while other patients may incur unnecessary tests and costs (under-specified results). However, the specificity and sensitivity are strong which indicates that the ABSST Short Form is able to differentiate patients who would likely benefit from a referral from those who would not.

Other questionnaires have been developed such as an 8-item screening tool to aid in identifying patients who may have OAB in a busy primary care setting which has a sensitivity of 98% and specificity of approximately 83% [32]. Moreover, the 3-Item OAB awareness tool (which is a short version of the 8-item screening tool) has recently been validated with a sensitivity of 82% and specificity of 91%. In addition, the International Prostate Symptom Score IPSS has been used to identify the severity of bladder symptoms in an MS population (All patients had an Expanded Disability Status Scale score of <6.5, with a mean of 3.4). The 8-item IPSS was originally developed to measure symptom severity in benign prostate hyperplasia. It has also been utilized to measure the prevalence of bladder problems over 2–3 years [33]. Only the ABSST Short Form has been developed or psychometrically tested to the scientific rigor of the FDA Guidance for PRO development. Overall the short form ABSST demonstrated good sensitivity and specificity as it has a positive predictive value of approximately 86% and a negative predictive value of approximately 93%. This shows that the short form ABSST maintains the integrity of the longer form tool which had a positive predictive value of approximately 76% and a negative predictive value of approximately 95% [16]. The sensitivity and specificity of the short form ABSST are in-line with other validated tools in the field and demonstrate its strength and potential positive impact in a clinic and primary care setting [34]. Of particular importance is the ability of the tool to detect more true-positive cases which increase the cost-effectiveness of screening and early detection of OAB problems as opposed to the false-positive cases, which can be detrimental to the screening process.

## Conclusions

The simplified scoring method of the ABSST allows for clinicians to easily identify patients with MS who may have potential urinary problems. The approach used to develop the scoring algorithm adheres to the FDA guidance, is psychometrically valid, and appropriately utilizes pivot anchoring [31], which has been widely used in the interpretation of clinically meaningful points along a categorical continuum. The methodology allowed for synthesis of meaningful cut-points where patients are likely to demonstrate urinary problems, as reported by clinicians. The reduction in items and scoring optimizes the most informational items for making sound clinical decisions. These data, when subjected to predictive validity calculations, indicate that clinicians can use the ABSST to refer patients appropriately. In conclusion, ABSST provides a new method for assessing bladder problems among MS patients, and may facilitate earlier diagnosis, treatment, and referral to a specialist.

## Abbreviations

ABSST: Actionable bladder symptom screening tool; FDA: Federal drug administration; IRB: Institutional review board; MS: Multiple sclerosis; NDO: Neurogenic detrusor overactivity; NPV: Negative predictive value; OAB: overactive bladder; OABqSF: Overactive bladder questionnaire – short form; PPV: Positive predictive value; PRO: Patient-reported outcomes; ROC: Receiver operating curve.

## Competing interests

Bates, D.: ‘The authors declare that they have no competing interests’.

Burks, J.: Consultant; Speaker’s Bureau for Acorda, Allergan (financed manuscript), Avanir, Bayer, Novartis, Sanofi-Aventis, and Serono. Consultant for Genzyme.

Globe, D.: Employed by Allergan (financed manuscript).

Signori, M.: Employed by Allergan (financed manuscript).

Hudgens, S.: Senior Lead on Research Study, funded by Allergan (financed manuscript).

Denys, P.: Advisor and investigator for Allergan (financed manuscript) and Ipsen, advisor and lecturer for Medtronic, Astellas, Coloplast and Astratech.

MacDiarmid, S.: Consultant; Speaker’s Bureau for Pfizer, Allergan (financed manuscript), Astellas, Uroplasty.

Nitti, V.: Astellas, Allergan (financed manuscript), Pfizer.

Odderson, I.: Speaker for Allergan (financed manuscript).

Perrin Ross, A: ‘The authors declare that they have no competing interests’.

Chancellor, M: Consultant and investigator, Allergan (financed manuscript).

## Authors’ contributions

SH and DG were involved in conducting the analyses and drafting the manuscript. JB, MC, SH, DG and MS were involved in developing the draft measures, developing the analysis plan and interpreting the results. All authors were involved in the design of the studies, review of the analysis and review, editing and approval of the manuscripts.

## Pre-publication history

The pre-publication history for this paper can be accessed here:

http://www.biomedcentral.com/1471-2377/13/78/prepub

## Supplementary Material

Additional file 1: Table S1 Actionable Bladder Symptom Screening Tool. Click here for file

## References

[B1] Compston A Coles A Multiple sclerosis Lancet 2002 359 1221 1231 10.1016/S0140-6736(02)08220-X 11955556

[B2] Noseworthy JH Lucchinetti C Rodriguez M Weinshenker BG Multiple sclerosis N Engl J Med 2000 343 938 952 10.1056/NEJM200009283431307 11006371

[B3] Shivane AG Chakrabarty A Multiple sclerosis and demyelination Curr Diagn Pathol 2007 13 193 202 10.1016/j.cdip.2007.04.003

[B4] National MS Ssociety About-multiple-sclerosis/What-we-know-about-ms/What-is-ms 2012 http://www.nationalmssociety.org/about-multiple-sclerosis/what-we-know-about-ms/what-is-ms/index.aspx

[B5] Courtney AM Treadaway K Remington G Frohman E Multiple sclerosis Med Clin North Am 2009 93 451 452x 10.1016/j.mcna.2008.09.014 19272518

[B6] Brady CM Dasgupta R Dalton C Wiseman OJ Berkley KJ Fowler CJ An open-label pilot study of cannabis-based extracts for bladder dysfunction in advanced multiple sclerosis Mult Scler 2004 10 425 433 10.1191/1352458504ms1063oa 15327041

[B7] De Seze M Ruffion A Denys P Joseph PA Perrouin-Verbe B The neurogenic bladder in multiple sclerosis: review of the literature and proposal of management guidelines Mult Scler 2007 13 915 928 10.1177/1352458506075651 17881401

[B8] Fowler CJ Panicker JN Drake M Harris C Harrison SC Kirby M A UK consensus on the management of the bladder in multiple sclerosis Postgrad Med J 2009 85 552 559 10.1136/jnnp.2008.159178 19789195

[B9] Dasgupta R Fowler CJ Bladder, bowel and sexual dysfunction in multiple sclerosis: management strategies Drugs 2003 63 153 166 10.2165/00003495-200363020-00003 12515563

[B10] Mahajan ST Patel PB Marrie RA Under treatment of overactive bladder symptoms in patients with multiple sclerosis: an ancillary analysis of the NARCOMS patient registry J Urol 2010 183 1432 1437 10.1016/j.juro.2009.12.029 20171697

[B11] Ghezzi A Carone R Del Popolo G Amato MP Bertolotto A Recommendations for the management of urinary disorders in multiple sclerosis: a consensus of the Italian multiple sclerosis study group Neurol Sci 2011 32 1223 1231 10.1007/s10072-011-0794-y 21948057

[B12] Dorman PJ Slattery J Farrell B Dennis MS Sandercock PA A randomised comparison of the EuroQol and short form-36 after stroke. United Kingdom collaborators in the International stroke trial BMJ 1997 315 461 10.1136/bmj.315.7106.461 9284664 PMC2127345

[B13] Hassan SJ Weymuller EA Jr Assessment of quality of life in head and neck cancer patients Head Neck 1993 15 485 496 10.1002/hed.2880150603 8253555

[B14] Iglesias C Torgerson D Does length of questionnaire matter? a randomised trial of response rates to a mailed questionnaire J Health Serv Res Policy 2000 5 219 221 11184958 10.1177/135581960000500406

[B15] Rolstad S Adler J Ryden A Response burden and questionnaire length: is shorter better? a review and meta-analysis Value Health 2011 14 1101 1108 10.1016/j.jval.2011.06.003 22152180

[B16] Burks J Chancellor M Bates D Denys P DeRidder D MacDiarmid S Development and validation of the actionable multiple sclerosis bladder health screening tool Int’L of MS Care 2013 24453782 10.7224/1537-2073.2012-049 PMC3883018

[B17] Nunnally J Bernstein I Psychometric theory 1994 3 New York: McGraw-Hill

[B18] Bond T Fox C Applying the Rasch Model: Fundamental Measurement in the Human Sciences 2001 New Jersey: Lawrence Earlbaum Associates

[B19] Streiner D Norman G Health Measurement Scales 2008 4 Oxford, England: Oxford University Press

[B20] Masters GN A rasch model for partial credit scoring Psychometrika 1982 47 149 174 10.1007/BF02296272

[B21] Campbell DT Fiske DW Convergent and discriminant validation by the multitrait-multimethod matrix Psychol Bull 1959 56 81 105 13634291

[B22] Linacre JM A User’s Guide to WINSTEPS 2006 Chicago: Mesa Press

[B23] Kuder GF Richardson MW The theory of the estimation of test reliability Psychometrika 1937 2 151 160 10.1007/BF02288391

[B24] Tape TG Interpreting Diagnostic Tests 2012 Omaha, Nebraska: University of Nebraska Medical Center

[B25] Patrick DL Burke LB Gwaltney CJ Leidy NK Martin ML Molsen E Content validity-establishing and reporting the evidence in newly developed patient-reported outcomes (PRO) Instruments for medical product evaluation: ISPOR PRO good research practices task force report: part 1-eliciting concepts for a new PRO instrument Value Health 2011 14 967 977 10.1016/j.jval.2011.06.014 22152165

[B26] Patrick DL Burke LB Gwaltney CJ Leidy NK Martin ML Molsen E Content validity-establishing and reporting the evidence in newly developed patient-reported outcomes (PRO) Instruments for medical product evaluation: ISPOR PRO good research practices task force report: part 1-eliciting concepts for a new PRO instrument Value Health 2011 14 978 988 10.1016/j.jval.2011.06.013 22152166

[B27] Litwin Mark S How to measure survey reliability and validity. Vol. 7 1995 Thousand Oaks: SAGE Publications, Incorporated

[B28] Diamond GA Forrester JS Analysis of probability as an aid in the clinical diagnosis of coronary-artery disease N Engl J Med 1979 300 1350 1358 10.1056/NEJM197906143002402 440357

[B29] Goldschlager N Use of the treadmill test in the diagnosis of coronary artery disease in patients with chest pain Ann Intern Med 1982 97 383 388 10.7326/0003-4819-97-3-383 7114636

[B30] Ransohoff DF Feinstein AR Problems of spectrum and bias in evaluating the efficacy of diagnosis tests N Engl J Med 1978 299 926 930 10.1056/NEJM197810262991705 692598

[B31] Bode RK Partial credit model and pivot anchoring J Appl Meas 2001 2 78 95 12000858

[B32] Coyne KS Margolis MK Zyczynski T Elinoff V Roberts R Validation of an Overactive Bladder Screener in a Primary Care Patient Population in the United States 2004 Poster presented at the 34th Joint Meeting of the International Continence Society and the International UroGynecological Association

[B33] Nortvedt MW Riise T Frugård J Mohn J Bakke A Skår AB Prevalence of bladder, bowel and sexual problems among multiple sclerosis patients two to five years after diagnosis Mult Scler 2007 13 106 112 10.1177/1352458506071210 17294618

[B34] Coyne KS Margolis MK Bavendam T Roberts R Elinoff V Validation of a 3-item OAB awareness tool Int J Clin Pract 2011 65 219 224 10.1111/j.1742-1241.2010.02561.x 21235701

