# Supplementary material for: Development of a short form and scoring algorithm from the validated actionable bladder symptom screening tool
Source: BMC Neurol. 2013 Jul 9;13:78. doi: 10.1186/1471-2377-13-78 (PMC3728236; doi:10.1186/1471-2377-13-78)
Supplement: Additional file 1: Table S1 — Actionable Bladder Symptom Screening Tool. [file 1471-2377-13-78-S1.doc]

**Actionable Bladder Symptom Screening Tool**

| INSTRUCTIONS: For the questions below, please check the box that best describes your bladder symptoms over the **past 7 days**. | | | | | | | |
| --- | --- | --- | --- | --- | --- | --- | --- |
| 1. In the past 7 days, during the day, how often did you feel that you had to urinate right away? | None of the time | Some of the time | | Most of the time | | All of the time | |
| 1. In the past 7 days, how often have you had urinary accidents/leakage? | None of the time | Some of the time | | Most of the time | | All of the time | |
| 1. In the past 7 days, during the day, how strong was the feeling that you needed to urinate right away? | Not at all strong | A little strong | | Moderately strong | | Extremely strong | |
| 1. In the past 7 nights, on a typical night, how often did you wake up in the night to urinate? | None of the time | One time | | Two times | | Three or more times | |
| 1. In the past 7 days, on a typical day, how many times did you urinate? | 0 – 3 times | 4 – 6 times | | 7 – 11 times | | 12 or more times | |
|  | | | | | | | |
| INSTRUCTIONS: For the questions below, please check the box that best describes impacts from bladder symptoms you may have experienced **recently**. | | | | | | | |
| 1. Recently, how much have your activities with friends and family been limited by your bladder problems? | Not at all | A little | | Moderately | | Extremely | |
| 1. Recently, how much has your ability to work (paid or volunteer) outside the home been limited by your bladder problems? | Not at all | A little | | Moderately | | Extremely | |
| 🞏 Does not apply | | | | | | | |
| 1. Recently, how embarrassed have you been because of your bladder symptoms? | Not at all | | A little | | Moderately | | Extremely |
|  | | | | | | | |
| 1. Would you like to receive help for your bladder problems? | | | | Yes  🞏 | | No  🞏 | |
